# Evaluation of emergency medical responses to nursing homes in a local area of Germany

**DOI:** 10.1186/s12873-025-01306-9

**Published:** 2025-08-05

**Authors:** Christine Gaik, Hinnerk Wulf, Valesco Mann, Dennis Humburg, Benjamin Vojnar

**Affiliations:** 1 Department of Anesthesiology and Intensive Care Medicine, University Hospital Giessen and Marburg, Campus Marburg and Philipps University of Marburg, Baldingerstraße, 35033 Marburg, Germany; 2 Department of Anaesthesiology, Intensive Care Medicine, and Pain Therapy, Agaplesion Evangelical Hospital Mittelhessen, Paul-Zipp-Straße 171, 35398 Giessen, Germany; 3 Department for Emergency Medicine, University Hospital Giessen and Marburg, Campus Marburg, Baldingerstraße, 35033 Marburg, Germany; 4 Department of Hazard Prevention and Emergency Service, District of Vogelsberg, Goldhelg 20, 36341 Lauterbach, Germany

**Keywords:** Ambulance, Emergency medical service, Injuries, Hip fracture, Nursing home

## Abstract

**Background:**

In rural areas with low population density, emergency medical services (EMS) are often the first source of acute medical care in nursing homes (NHs) when nursing staff are unable to manage the situation, and no specialist practitioner is immediately available. This study analysed EMS responses to NHs in a German region to evaluate their frequency, causes, and outcomes.

**Methods:**

This cross-sectional study analysed EMS responses to NHs in a German region from July 2020 to December 2024. Prehospital patient care was assessed using the ABCDE approach, with additional analysis of feedback codes transmitted to the control centre. Data were presented through descriptive statistics.

**Results:**

Among 81,727 EMS responses, 55,900 were acute emergencies, including 5,738 cases to NHs. The median age of NH patients was 84 years (IQR 78–89), with 41% male and 59% female. Spontaneous breathing was unremarkable in 72% (4,141/5,738) of cases. A pulse rate of 60–100 beats per minute was recorded in 71% (4,093/5,738), and systolic blood pressure ranged between 100 and 140 mmHg in 45% (2,572/5,738). Neurologically, 80% (4,570/5,738) were classified as ‘alert’, and 12% (674/5,738) as ‘responsive to speech’. The most common diagnoses included fractures (face, head, extremities) and pneumonia. In 81.6% of cases, disturbances in all vital signs were classified as ‘low’ to ‘moderate’.

**Conclusion:**

Most EMS responses to NHs involved patients with stable vital signs, and critically acute conditions were rare. Hospital admissions were predominantly ‘non-urgent’, with patients mainly transferred to primary care hospitals, which typically offer basic medical services, suggesting lower medical complexity. Falls and related injuries, particularly suspected hip fractures, were the leading reason for EMS utilisation. These findings highlight the need for targeted strategies to enhance the management of ‘non-urgent’ cases within NHs and to reduce avoidable hospital admissions, which may partly result from limited access to medical support.

**Trial registration:**

This study was registered in the German Clinical Trials Register on 17 December 2024 (ID DRKS00035675).

## Background

Over the coming years, the demand for professional nursing and elderly care is expected to increase [1]. Due to higher life expectancy and falling birth rates, the proportion of the ageing population is growing rapidly in many countries [2]. According to a forecast by the German Federal Statistical Office, the proportion of people aged ≥ 65 years in Germany is projected to rise from 22.1% in 2022 to approximately 29.7% by 2070 [3]. The projected growth of the elderly population is mainly driven by the aging of the post-war “baby boomer” generation, while at the same time, consistently low birth rates have led to a shrinking younger population [4]. This trend is also likely to lead to an increased demand for nursing homes (NHs).

NH residents often present with pre-existing multimorbidity, including higher rates of dementia and severe mental disorders that require intensive medical care [1, 5]. The complexity of different comorbidities is a major challenge for medical staff [6].

The widespread shortage of nursing staff, general practitioners and specialist physicians, particularly in low-population areas, often leaves local emergency medical services (EMS) as the only available option for timely medical assistance [7]. This situation is expected to contribute to a rise in hospital admissions among older patients [8, 9]. In recent years, ambulance services have already reported an increase in emergency calls from NHs [10, 11].

There are only a few studies investigating EMS use in NHs. However, existing data suggest that older adults (≥ 65 years) have significantly higher EMS utilisation rates than younger individuals, with emergency physician involvement being particularly frequent among NH residents, indicating elevated prehospital care needs in this population [12]. Furthermore, studies indicate that more than half of the emergency department (ED) visits by NH residents could potentially be avoided [13]. This study aimed to analyse emergency service deployments to NHs over a period of 4.5 years. The primary objectives were to identify the most common medical conditions leading to emergency calls, evaluate the outcomes of the priority-based assessment using the ABCDE approach, and assess how critically EMS staff perceived the overall situation.

## Methods

### Ethics approval and setting

This cross-sectional study was conducted in the Department of Anaesthesiology and Intensive Care Medicine at Marburg University Hospital, Germany (German Clinical Trials Register ID: DRKS00035675; registered on 17 December 2024), with ethics approval obtained from the Ethics Committee of the Medical Faculty, Philipps University of Marburg (AZ 24-287-RS), and in accordance with the Declaration of Helsinki. As this was a retrospective analysis of anonymised data, individual consent was not required. We analysed all EMS responses to NHs in a local area of Germany from July 2020 to December 2024. The objective was to assess the frequency, causes, and outcomes of these deployments, as well as to evaluate the level of urgency required for managing of each patient and their specific medical condition. This manuscript adheres to the current “Strengthening the Reporting of Observational Studies in Epidemiology” (STROBE) guidelines.

### Study population

For this study, a county in Germany with an above-average ageing population was selected: Vogelsbergkreis in Hessen, Germany. Located north of Frankfurt, near the geographical centre of Germany, Vogelsbergkreis covers an area of approximately 1,460 square kilometres (563 square miles). The district consists of 19 municipalities, including 7 towns [14]. Vogelsbergkreis has a population of approximately 106,792 residents [15]. With a population density of about 73 people per square kilometre, it is significantly lower than the national average of 273 people per square kilometre [16]. Approximately 25% of the population is aged 65 years or older, and the median age is 47 years [17].

### EMS system and hospital infrastructure in the study region

Previous studies have demonstrated substantial heterogeneity in prehospital EMS across Europe [18]. In some countries, physicians play a key role in emergency care, whereas in others, this role is carried out by specialised non-physician professionals, such as paramedics [19]. In Germany, emergency dispatch centers determine whether an emergency physician is required at the scene based on the details provided in the emergency call. Ambulances are staffed with emergency medical personnel, including advanced paramedics and/or emergency medical technicians (EMTs).

Advanced paramedics (“Notfallsanitäter”) in Germany represent the highest non-physician qualification and complete a three-year training program. Although trained in advanced prehospital care, the application of many medications, including opioids such as fentanyl and cardiovascular agents, remain restricted to physicians. Advanced paramedics may independently administer a limited number of life-saving drugs (e.g., intravenous or intramuscular adrenaline) in well-defined emergency situations, while basic interventions such as oxygen or electrolyte administration may also be performed by other EMS personnel [20]. An additional physician-staffed ground or air-based emergency unit can typically be dispatched at any time if needed. However, delays in dispatching and awaiting a physician-staffed emergency unit, particularly in rural areas with extended travel distances, may contribute to prolonged on-scene times.

In Germany, hospitals are commonly categorised into primary, secondary, and tertiary care hospitals based on their bed capacity, range of specialties, and service scope. Primary care hospitals offer basic inpatient services, mainly internal medicine and general surgery, and are often located in rural areas. Secondary care hospitals provide a broader range of specialties and serve as regional referral centers. Tertiary care hospitals, including university hospitals, offer highly specialised and complex services and are involved in teaching and research [21].

### Study design

In the selected county, all emergency service teams use digital patient data management systems to document patient care. In the digital documentation system (NIDApad, medDV GmbH, Fernwald, Germany), EMS personnel select the deployment location from a predefined list (e.g., private residence, workplace, school, NH). Only EMS responses explicitly documented with the deployment location “nursing home” in the digital system were included.

### Data collection and statistical analysis

All prehospital patient care reports were documented using tablets (NIDApad) and electonically transmitted to a secure server. A fully anonymised copy of these patient records, excluding any free-text medical history, was accessed via a secure VPN connection. Cases with duplicate documentation by both ambulance and emergency physician teams were excluded to avoid double counting (*n* = 775), as outlined in the results section. Routine data were analysed using NIDAanalyse software (Version 1.5.38.138, medDV GmbH, Fernwald, Germany). The reports included detailed information on primary symptoms, vital signs, and medical interventions.

In addition, a feedback code was transmitted to the receiving hospital before each transport, categorising the type and severity of the condition based on the extended ABCDE approach [22, 23]. The severity of vital signs was assessed using a five-level code ranging from 1 (very low) to 5 (critical) for each domain (e.g., breathing, circulation, consciousness). This coding reflects the overall clinical impression based on multiple parameters (e.g., respiratory rate, breathing pattern, and respiratory effort). A score of “1 – Very low” indicates minimal or no impairment of the respective physiological system, whereas “5 – Critical” reflects a severely compromised condition.

In addition, the most important vital signs were evaluated in detail and presented descriptively based on the available data. Abnormal vital signs were defined as follows: pulse rate < 60 or > 100 beats per minute (bpm), systolic blood pressure < 100 or > 140 mmHg, respiratory rate < 12 or > 20 breaths per minute, or oxygen saturation < 96% [24, 25].

Missing values were transparently reported for each variable in the results section. The total number of cases reflects the complete available dataset within the defined period and setting. Continuous data are presented as means with standard deviation (SD), median, and interquartile range (IQR), while categorical data are reported as absolute numbers and proportions. Statistical analyses were performed using Excel 2013 (Microsoft, Redmond, Washington, USA). As this retrospective observational study included all EMS responses from July 2020 to December 2024, no formal sample size calculation was conducted.

## Results

### Public health

In the selected local district, 24 NHs and 72 general practitioners operating at approximately 48 different practice locations, alongside four hospitals. Covering an area of 1,460 square kilometres (563 square miles), EMS are supported by 14 ambulances and three emergency physician vehicles, which are stationed at 12 locations.

### Number of emergency medical responses

**Fig. 1:**
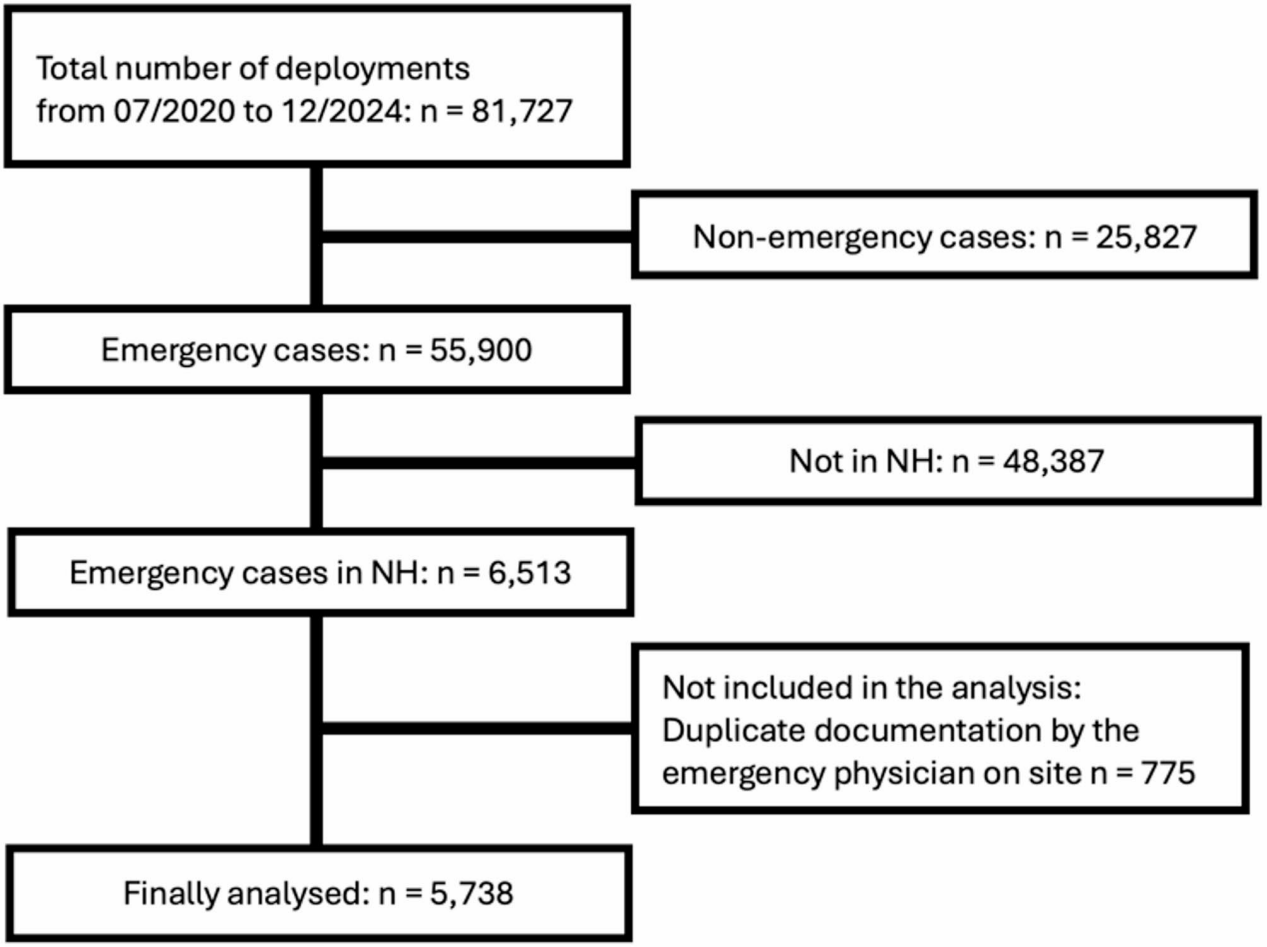
Flowchart of analysis process from total deployments (*n* = 81,727) to the final dataset (*n* = 5,738)

Of the 81,727 emergency medical responses analysed from July 2020 to December 2024, 55,900 were classified as emergencies, while the remaining 25,827 were categorised as ‘non-emergency patient transports’. Among the 55,900 emergency responses, 6,513 occurred as responses to NHs. 775 cases with separate documentation by dispatched emergency physicians were excluded from the analysis (duplicate documentation), resulting in a final dataset of 5,738 emergency transfers (Fig. 1).

### Statistics on emergency responses

**Fig. 2:**
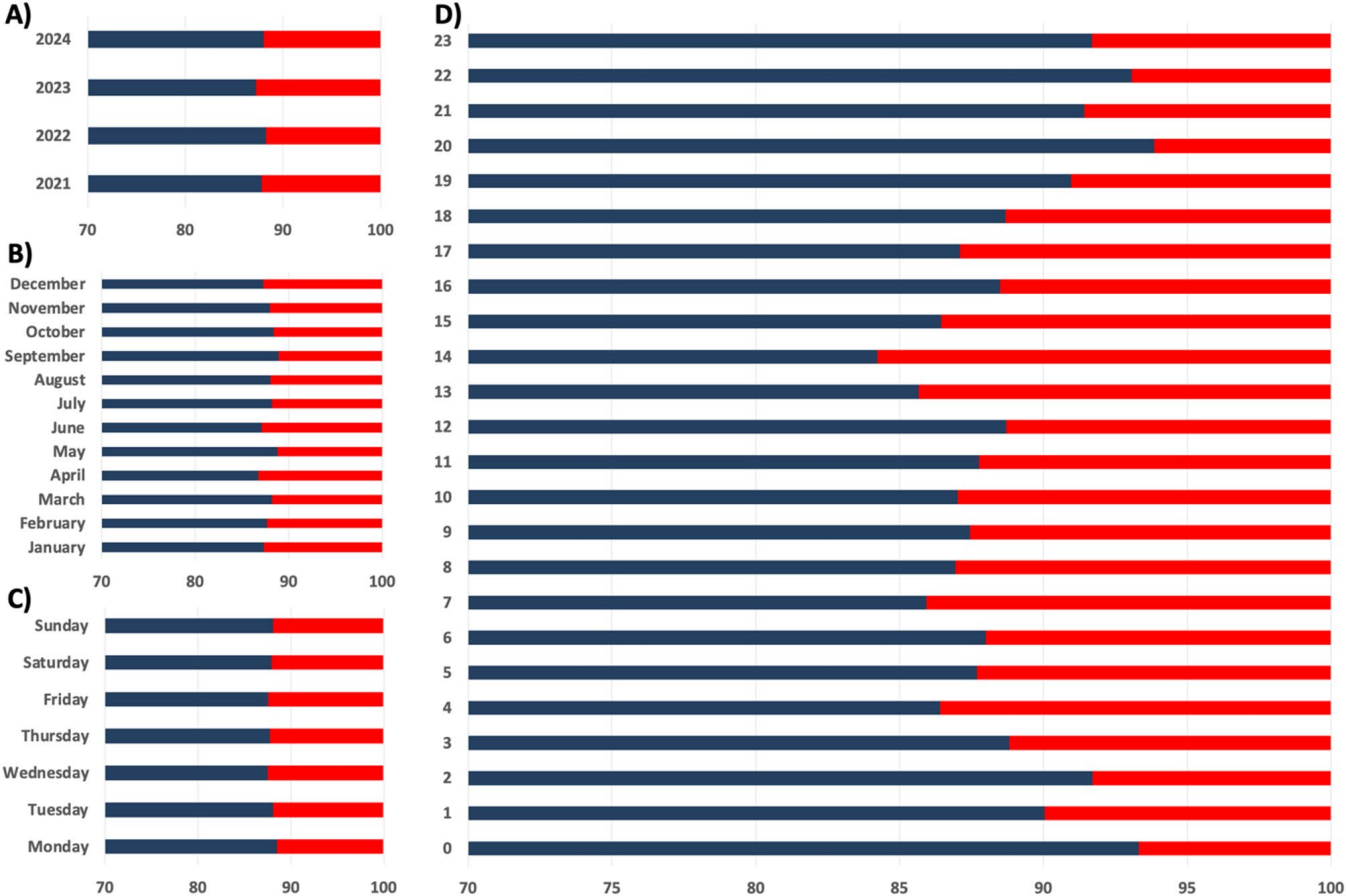
This figure illustrates the distribution of emergency service responses to NHs (red bars) compared to those occurring outside NHs (blue bars). It shows the relative proportions of these responses over several dimensions. (**A**) Annual Trends, (**B**) Monthly Trends, (**C**) Weekly Trends, (**D**) Hourly Distribution. The figure highlights the proportion of emergency responses to NHs relative to the total number of responses, emphasising the contribution of NH calls to the overall emergency workload. The data cover the period from July 2020 to December 2024

Overall, approximately 12% of all EMS deployments (5,738/55,900) were emergency responses to NHs. Monthly data indicates slight seasonal variations, with a higher proportion of NH deployments observed in April and June. Across all weekdays, NH deployments consistently accounted for approximately 11–12% of total cases. The distribution of EMS deployments by year, month, weekday, and time of day, is shown in Fig. 2. The median travel time to a NH after call acceptance was six minutes (IQR 3–11). The median duration of on-site emergency medical care, measured from arrival to departure, was 27 min (IQR 20–35). Among hospital transports, 13% were conducted with lights and sirens activated, while 87% were proceeded without. Hospital assignments were based on feedback codes reflecting clinical severity, in combination with real-time hospital availability as coordinated by the regional emergency dispatch center. A total of 5,539 emergency transfers were made from NHs to nearby hospitals. Most patients were transported to Eichhof Hospital Lauterbach, a primary care hospital with 240 beds, which handled 2,362 cases (43%). This was followed by Alsfeld Hospital, another primary care hospital with 154 beds, which recorded 1,289 transfers (23%). A tertiary care hospital like Fulda Clinic (1,000 beds), managed 407 cases (7%), while the University Hospital Giessen and Marburg, Marburg site (1,140 beds), received 181 cases (3%). Schlüchtern Hospital, a secondary care hospital with 276 beds, recorded 175 cases (3%). The remaining 1,125 transfers (20%) were distributed among various hospitals in neighbouring districts. In 199 cases, the receiving hospital was not recorded and therefore not included in the hospital-level analysis.

### Initial findings on site

For emergency responses to NHs, the median patient age was 84 years (IQR 78–89). Among these patients 41% were men and 59% were women. For emergency responses outside NHs, the median patient age was 60.5 years (IQR 44–81). In this group, 51% were men and 49% were women.

**A-Airway**

No patient report indicated a case of complete airway obstruction.

**B-Breathing** (oxygen saturation, respiratory rate, auscultation findings)

In 72% (4141/5738) of cases, patients exhibited unremarkable spontaneous breathing. Dyspnoea was reported in 21% (1183/5738), while 2% (89/5738) presented with crackles (alveolar rales). Hyperventilation was documented in 1% (36/5738), cyanosis in 1% (41/5738), and apnea in 1% (30/5738) of cases. The remaining 2% (120/5738) displayed other respiratory findings. Information regarding respiratory status (B problem) was missing in 2% (98/5738) of reports.

A respiratory rate below 12 breaths per minute was recorded in 4% (216/5738) of patients, while 76% (4349/5738) had a rate between 12 and 20 breaths per minute. A respiratory rate of 21 or more breaths per minute was observed in 14% (792/5738). The median respiratory rate was 15 breaths per minute (IQR 14–18). Respiratory rate was missing in 7% (381/5738) of reports.

The data set for peripheral oxygen saturation was faulty/incomplete and could not be analysed.

**C-Circulation** (capillary refill time, pulse rate, systolic blood pressure)

Shock was documented in 3% (177/5738) of cases, while severe bleeding was recorded in 1% (80/5738) of patients.

Capillary refill time exceeded two seconds in 17% (974/5738) of patients, while 82% (4670/5738) had a refill time under two seconds. This information was missing in 2% (94/5738) of reports.

A recorded pulse rate of fewer than 60 bpm was observed in 8% (434/5738) of patients. The pulse rate ranged between 60 and 100 bpm in 71% (4093/5738), between 101 and 140 bpm in 15% (837/5738), and exceeded 140 bpm in 1% (55/5738) of cases. The median pulse rate was 80 bpm (IQR 70–94). Pulse rate data were missing in 6% (319/5738) of reports.

Systolic blood pressure was below 100 mmHg in 10% (592/5738) of patients, between 100 and 140 mmHg in 45% (2572/5738), and above 140 mmHg in 33% (1906/5738). The median systolic blood pressure was 131 mmHg (IQR 112.0-153.0 mmHg). Systolic blood pressure data were missing in 12% (668/5738) of reports.

**D-Disability** (neurological status assessed using the AVPU scale, blood glucose and pain score)

In 80% (4570/5738) of cases, the neurological status was described as ‘alert’, in 12% (674/5738) as ‘responsive to speech’, in 5% (274/5738) as ‘responsive to pain’, and in 2% (93/5738) as ‘unresponsive’. The remaining 2% (127/5738) of reports documented ‘other’ neurological findings.

A blood glucose level below 40 mg/dl was recorded in 0.3% (17/5738) of cases, while 2% (90/5738) had values between 41 and 80 mg/dl. A blood glucose range of 81 to 160 mg/dl was documented in 32% (1861/5738), and levels exceeding 160 mg/dl were noted in 17% (999/5738). The median blood glucose level was 138 mg/dl (IQR 113.0–180.0 mg/dl). No blood glucose measurements were recorded for 48% (2771/5738) of patients.

Pain was assessed using the Numeric Rating Scale (NRS), a standardised tool ranging from 0 (no pain) to 10 (worst possible pain). A pain score of five or higher on the NRS was documented in 7% (392/5738) of NH emergency responses. A NRS score between 0 and 4 was recorded in 81% (4660/5738) of cases. Pain score data were missing in 12% (686/5738) of reports.

**E-Exposure**

The skin condition was documented as ‘unremarkable’ in 49% (2806/5738) of patients. ‘Reduced skin turgor’ was noted in 35% (2022/5738), ‘peripheral oedema’ in 4% (231/5738), and ‘cold sweat’ in 3% (152/5738). No skin condition was recorded for 9% (527/5738) of patients.

### Feedback code – type and severity of the condition

The most common feedback codes included fractures of the face, head, and extremities and pneumonia (see Table 1). Among suspected fractures, femoral neck fractures were one of the most frequently documented diagnoses, with the emergency team recording this suspected diagnosis. Of all patients with this suspected diagnosis, 33% were male and 67% were female. In 81.6% of cases, disturbances across all categories – breathing, cardiovascular system, consciousness levels, neurological deficits, injuries, and pain – were classified as ‘very low’ to ‘moderate’. In 13.7% of cases, one category was rated as ‘severe’ or ‘critical’, while in 4.6% of cases, two categories were classified as ‘severe’ or ‘critical’.

Regarding hospital admissions, 4% (210/5738) of emergency cases were classified as non-urgent. In 11% (649/5738) of cases, transportation to the hospital was performed for outpatient treatment or diagnostic exclusion. In 75% (4315/5738) of cases, patients were admitted for inpatient care, while 10% (562/5738) required immediate admission for emergency treatment (e.g., trauma bay or stroke unit). The remaining 0.04% (2/5738) of cases contained invalid values.

**Table 1:** A feedback code was created for all 5,738 patients following the initial assessment, summarising the patient’s condition and severity. This table provides an overview of the five most common feedback codes/diagnoses and the severity levels of all documented vital signs, including breathing, cardiovascular system, levels of consciousness, neurological deficits, injuries, and pain. The data are based on the feedback codes of all emergency protocols involving NH residents and are presented in total numbers and percentages. Variations in total numbers for the severity levels of vital signs are due to missing data or incomplete documentation

**Five most common feedback codes:**	
Facial/Head Injury	512 (9%)
Closed Extremity Injury	333 (6%)
Bronchitis/Pneumonia	330 (6%)
Nonspecific Symptoms	309 (5%)
Stroke/TIA/Bleeding < 6 h	224 (4%)
**Severity of vital signs based on analysed feedback codes (n)**	
Breathing (*n* = 5377)	
Very low	3493 (65%)
Mild	1212 (23%)
Moderate	282 (5%)
Severe	361 (7%)
Critical	29 (1%)
**Cardiovascular System (** ***n*** ** = 5379)**	
Very low	2784 (52%)
Mild	2024 (38%)
Moderate	361 (7%)
Severe	183 (3%)
Critical	27 (1%)
**Level of Consciousness (** ***n*** ** = 5376)**	
Very low	3961 (74%)
Mild	1173 (22%)
Moderate	102 (2%)
Severe	81 (2%)
Critical	59 (1%)
**Neurological Deficits (** ***n*** ** = 5372)**	
Very low	2720 (51%)
Mild	2149 (40%)
Moderate	177 (3%)
Severe	311 (6%)
Critical	15 (0%)
**Injuries (** ***n*** ** = 5379)**	
Very low	3650 (68%)
Mild	967 (18%)
Moderate	593 (11%)
Severe	168 (3%)
Critical	1 (0%)
**Pain (** ***n*** ** = 5377)**	
Very low	3226 (60%)
Mild	1448 (27%)
Moderate	606 (11%)
Severe	92 (2%)
Critical	5 (0%)

TIA = Transient Ischemic Attack

## Discussion

We analysed all EMS responses to NHs in a local area in central Germany from July 2020 to December 2024 to evaluate the frequency, causes, and outcomes of these deployments. Initial on-site assessments indicated that the majority of patients exhibited stable respiratory and haemodynamic status. Mild deviations, such as slight respiratory distress, irregular respiratory rates, and blood pressure variability, were observed in a small proportion of patients. Most patients were neurologically alert, with notable cases of hypo/hyperglycaemia being rare. Overall, the ABCDE assessment revealed that major physiological disturbances were uncommon, and most patients maintained stable clinical parameters across all domains. Analysis of the feedback code sent to the emergency call centre indicated that many emergency responses to NHs did not involve acute, life-threatening conditions, but rather medically non-urgent situations.

### Distribution of EMS deployments

The hourly analysis revealed dynamic patterns in emergency call distribution. During early morning hours, over 90% of calls originated from outside NHs, while the proportion of NH-related calls peaked at 16% in the afternoon, reflecting typical daily activity cycles. Patients involved in emergency responses were significantly older than those outside NHs. Additionally, a higher proportion of women were observed in NH emergencies compared to non-NH-emergencies. This gender distribution likely reflects the higher life expectancy of women [24], resulting in a greater proportion of female residents in NHs. This finding suggests that NH residents in our study population represent an older and more predominantly female patient group in emergency care. Evidence from previous studies supports the increased risk of emergency admissions among older individuals [25–27].

### ABCDE approach

None of the reports documented a complete airway obstruction, indicating the absence of acute or life-threatening airway compromise. While milde dyspnoea was common, severe respiratory complications were relatively rare, and most patients had stable respiratory parameters. A small subset experienced mild ventilatory disturbances, which are in line with findings reported by Kauppi et al. (2020), who associated such patterns with chronic conditions such as chronic obstructive pulmonary disease (COPD) or heart failure [28].

However, this could not be conclusively determined, as we were unable to analyse the free-text medical history, including long-term medication use, due to data protection regulations.

In addition to suspected fractures, bronchitis and pneumonia ranked among the top five conditions leading to EMS alerts in our study. Studies investigating strategies to reduce ED admissions have assessed various interventions [29, 30]. Care pathways for lower respiratory tract infections and influenza vaccination represent two key options within these approaches [31]. Whether such interventions also contribute to a reduction in emergency calls and EMS deployments remains unclear, as the available studies primarily focus on hospital admissions and do not provide data on EMS utilization.

A physiological pulse rate was recorded in most patients, with bradycardia or tachycardia occurring in a minority. Systolic blood pressure was largely within normal limits, with hypotension being rare, while hypertension likely reflected underlying chronic conditions. Signs of circulatory instability, such as oedema or cold sweat, were documented infrequently, suggesting that severe cardiovascular compromise was uncommon.

Previous research suggests that chronic neurological disorders, such as dementia and Parkinson’s disease, more frequently lead to hospital transport, whereas acute neurological events are rare [32]. Although this study did not specifically examine this aspect, the rare documentation of severe neurological impairments in our data appears to support this observation. Accordingly, mild neurological deficits were documented in approximately 40% of patients, further emphasising the importance of chronic disease in this population. Overall, the findings suggest a low prevalence of acute, life-threatening neurological and metabolic disturbances. A large proportion of patients were transported to primary care hospitals, underscoring the essential role of these facilities in managing emergencies and suggesting that many patients presented with less complex medical conditions. Only a limited number of patients were transferred to a tertiary care hospitals, indicating that relatively few cases required highly specialised medical treatment.

### Falls in NHs

Our analysis indicates that falls, particularly those resulting in fractures, are among the most common reasons for emergency responses in NHs. This observation aligns with the findings by Pulst et al., who reported that over 33% of unplanned hospital transfers are due to falls, with many of these transfers occurring without prior medical consultation [33]. Similar conclusions were reached by the study groups of Gruneir et al. and Guerbaai et al. [34, 35]. Our study further highlights that femoral neck fractures are among the most frequently suspected fractures caused by falls is, with two-thirds of these cases occurring in women. These results are consistent with the findings of Labaka et al., who demonstrated that femoral fractures are more prevalent in women and frequently lead to ED admission [25]. Several factors may contribute to the high number of falls in elderly patients, including medication side effects, cognitive impairment, frailty, and vision and hearing deficits [36, 37]. While physical restraints are sometimes considered a preventive strategy against falls in nursing homes, current evidence remains inconclusive regarding their effectiveness and highlights the need for further research [38].

### Pain management

The most common injuries following falls in the NHs were facial and head injuries, femoral neck fractures, and closed limb injuries. Despite the severity of these injuries, over 82% of patients reported an initial NRS score between 0 and 4. Data on long-term analgesic use were not available and, therefore, could not be included in our analysis. The overall relatively low reported pain scores might reflect regular use of analgesics in this population [39]; however, this remains speculative, as data on long-term medication were not available. Several studies have examined the adequacy of prehospital pain management in older patients with fractures, even among those receiving regular analgesic therapy [40–42]. Pain may still be undertreated in the prehospital setting, particularly in elderly patients. Contributing factors include limited initial pain assessment, absence of reevaluation during transport, and overall insufficient treatment [40, 41, 43]. In cases presenting with low pain intensity, regional anaesthesia may be a viable option for managing proximal hip fractures during transport, particularly in multimorbid patients on polypharmacy, as it could help to minimise opioid use. While this approach is not yet widely adopted in Europe, it could be a valuable addition to paramedic training programs [44].

### Feedback code – type and severity of the condition

The analysis of the feedback code sent to the emergency call centre indicates that many emergency responses to NHs did not involve acute, life-threatening conditions, but rather medically non-urgent situations. This is further supported by the low proportion of transports conducted with lights and sirens activated. However, such emergency calls should not automatically be considered avoidable, as nursing staff initiating the call are often not able to determine whether the situation qualifies as an acute, life-threatening condition. Notably, the feedback code also revealed that three out of four of these patients were ultimately admitted for inpatient treatment. Although our data do not provide insight into the exact reasons for admission, structural challenges - such as limited on-site diagnostic capabilities, insufficient general practitioners availability, and the complexity of care - may have played a contributing role. This aligns with studies analysing ED visits by NH residents and health insurance data, which report similar trends [35, 45, 46]. Many transfers are not due to medical emergencies but rather the inability of NHs to adequately manage patients with multiple chronic conditions, often due to limited on-site resources and the unavailability of general practitioners for timely home visits [47]. The combination of staff shortages and high workloads can create challenges in meeting the complex care needs of residents [33, 47]. Potential improvements include enhanced communication between general practitioners and nursing staff, increased staffing levels, and improved training for NH personnel [48]. International comparisons indicate significant differences in the training and qualifications of NH staff. For instance, Fassmer et al. found that residents of German NHs are hospitalised more frequently than their Dutch counterparts [49]. This discrepancy has been linked to the higher qualification levels of NH staff in the Netherlands, where regular interdisciplinary training specifically focuses on managing patients with multiple chronic conditions and complex care needs [33, 49].

Pulst et al. demonstrated that general practitioners are involved in fewer than 35% of unplanned hospital transfers of NH residents, leading to potentially avoidable admissions [33]. Similarly, Gruneir et al. highlighted that in many cases, no medical consultation occurs before hospitalisation, even during regular working hours, despite the availability of physicians [34]. Both studies emphasise the need for better involvement of general practitioners and closer inter-professional collaboration to reduce unnecessary hospital transfers and strengthen on-site care. In this context, targeted training of nursing staff and closer cooperation with external medical facilities, such as radiology practices, could be particularly beneficial [33, 34].

Strengthening partnerships with external health care providers, including community health centres and primary care centres, may contribute to a more efficient, patient-centred approach while reducing the burden on hospitals [50].

While our dataset did not include facility-level data on NH staffing or nurse qualifications, the pattern of low-acuity EMS responses and hospital admissions despite stable vital signs suggests structural limitations in long-term care. Although not directly measurable in this study, such factors are well documented in the literature as key drivers of potentially avoidable transfers from NHs. Our interpretation in this regard is therefore hypothetical but supported by consistent international evidence [33, 35, 45–47, 49]. This contextualization supports a meaningful interpretation of EMS utilization patterns and may inform future research incorporating care facility characteristics.

### Strengths and limitations

We selected a region with a high proportion of elderly residents, as such areas typically have an increased density of NHs. Additionally, mostly rural regions often face limited healthcare infrastructure, posing specific challenges for prehospital emergency care provided to NH residents. This study analysed 5,738 emergency responses involving NH residents, selected from a comprehensive dataset of 81,727 EMS reports. The dataset included all emergency medical responses in the region, allowing for a detailed assessment of the frequency, causes, and outcomes of EMS visits in NHs, with particular attention to case urgency. However, several limitations should be considered when interpreting the results. First, the analyses are based on a database that may contain incomplete or inaccurate data, potentially affecting the reliability of certain comparisons and limiting the external validity of the findings. Second, due to data protection regulations, handwritten medical history records were not included, which may have led to omission of relevant clinical information. Third, we did not receive feedback from the hospitals or EDs regarding whether the admissions were clinically justified or whether the suspected diagnoses were confirmed. Despite these limitations, our findings provide valuable insights into emergency care in NHs and establish a solid foundation for future research in this field.

## Conclusion

Our study demonstrates that during most EMS responses in NHs, patients exhibited predominantly stable vital functions, with serious acute medical conditions observed only in rare cases. The analysis of feedback codes from the scene to the control centre revealed that the majority of EMS requests were not classified as acutely urgent. Nevertheless, a significant proportion of patients were transported to the hospital, indicating potential deficiencies in on-site care. This finding aligns with the observation that most patients were transferred to primary care facilities, suggesting that many presented with less complex medical conditions, while only a small number required referral to specialised centres.

Falls were the most common reason for EMS activation and frequently resulted in suspected femoral neck fractures, particularly among female residents. Reported pain levels in patients with fall-related injuries were generally low, potentially due to long-term analgesic therapy. These findings highlight the need for targeted interventions aimed at improving the management of less urgent cases within NHs and reducing avoidable hospital admissions.

## Data Availability

The data supporting the findings of this study are available from the authors; however, access is restricted as they were used with permission from the Center for Hazard Prevention, 36341 Lauterbach, Germany, for this study and are not publicly available. Data may be obtained from the authors upon reasonable request and with approval from both the Center for Hazard Prevention and the Medical Director of Emergency Medical Services.

## Abbreviations

COPD: Chronic Obstructive Pulmonary Disease
ED: Emergency Department
EMS: Emergency Medical Services
EMTs: Emergency Medical Technicians
IQR: Interquartile Range
NRS: Numerical Rating Scale
NH(s): Nursing Home(s)
SD: Standard Deviation
STROBE: Strengthening the Reporting of Observational Studies in Epidemiology
TIA: Transient Ischemic Attack
VPN: Virtual Private Network
